# Biopharming of Lactoferrin: Current Strategies and Future Prospects

**DOI:** 10.3390/pharmaceutics17081023

**Published:** 2025-08-07

**Authors:** Rajaravindra Konadaka Sri, Parthasarathi Balasamudram Chandrasekhar, Architha Sirisilla, Qudrathulla Khan Quadri Mohammed, Thejasri Jakkoju, Rajith Reddy Bheemreddy, Tarun Kumar Bhattacharya, Rajkumar Ullengala, Rudra Nath Chatterjee

**Affiliations:** 1 ICAR-Directorate of Poultry Research, Hyderabad 500030, India; parthb763@gmail.com (P.B.C.); archithasirisilla@gmail.com (A.S.); ullengala@yahoo.com (R.U.);; 2 ICAR-National Research Centre on Equines, Hisar 125001, India

**Keywords:** biopharming, lactoferrin, microbial expression, therapeutic protein expression, mammalian cell line, transgenic animals, transgenic crops, production strategies

## Abstract

Lactoferrin (LF) is an 80 kDa iron-binding glycoprotein primarily found in milk, saliva, tears, and nasal secretions. LF is well known for its antibacterial and immunomodulatory effects. However, the extraction of LF from milk is inadequate for large-scale therapeutic applications, presenting a challenge for economic mass production. Recombinant protein expression systems offer a solution to overcome this challenge and efficient production of LF. This review discusses recent progress in the translational research of LF gene transfer and biopharming, focusing on different expression systems such as bacteria, yeast, filamentous fungi, transgenic crops, and animals as well as purification methods. The optimization of expression yields, prospects for genetic engineering, and biotechnology to enhance LF production for biomedical applications are emphasized. This review systematically sourced the literature from 1987 to 2025 from leading scientific databases, including PubMed, Scopus, Web of Science, and Google Scholar. Despite ongoing debates, progress in this field indicates a viable path towards the effective use of LF in therapeutic settings.

## 1. Introduction

**Figure 1:**
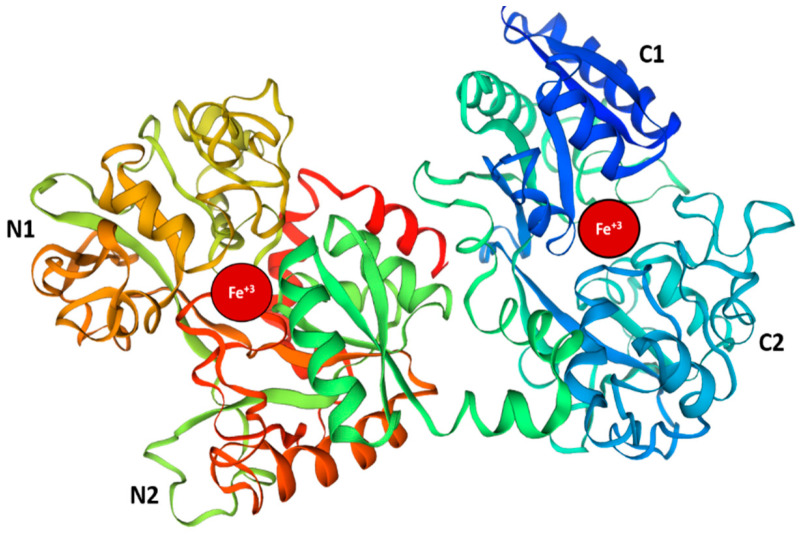
Three-dimensional (3D) structure of human lactoferrin protein. The three-dimensional structure of the human lactoferrin (hLF) protein reveals two globular shapes that are similar in form and symmetry, labeled as the N and C lobes. Each lobe is further divided into two subdomains: N1 and N2 for the N lobe, and C1 and C2 for the C lobe. The iron ions are represented as red spheres.

Lactoferrin (LF) is a glycoprotein commonly found in milk, saliva, tears, serum, mucosal secretions, and neutrophils. It has high affinity for binding with iron and consists of 601 to 700 amino acids, with a relative molecular mass of approximately 80 kDa. Its secondary structure is characterized by α-helices, β-pleated sheets, and turns [1,2]. LF is composed of two globular lobes, each capable of binding one iron ion (Fe^3+^) in a deep cleft (Figure 1). These lobes are connected by a three-turn α-helix, which is further divided into two subdomains: N1 and N2; C1 and C2 [3,4,5]. LF can be classified into three forms based on its saturation level: apo-lactoferrin (no Fe^3+^ ion), mono-ferric (one Fe^3+^ ion), and holo-lactoferrin (two Fe^3+^ ions) [6]. Apo-lactoferrin plays a crucial role in protecting against pathogenic microbes and modulating immune responses [7], while holo-lactoferrin is essential for transporting iron to support normal cellular functions. Maintaining a balance between these forms is vital for health and homeostasis.

**Figure 2:**
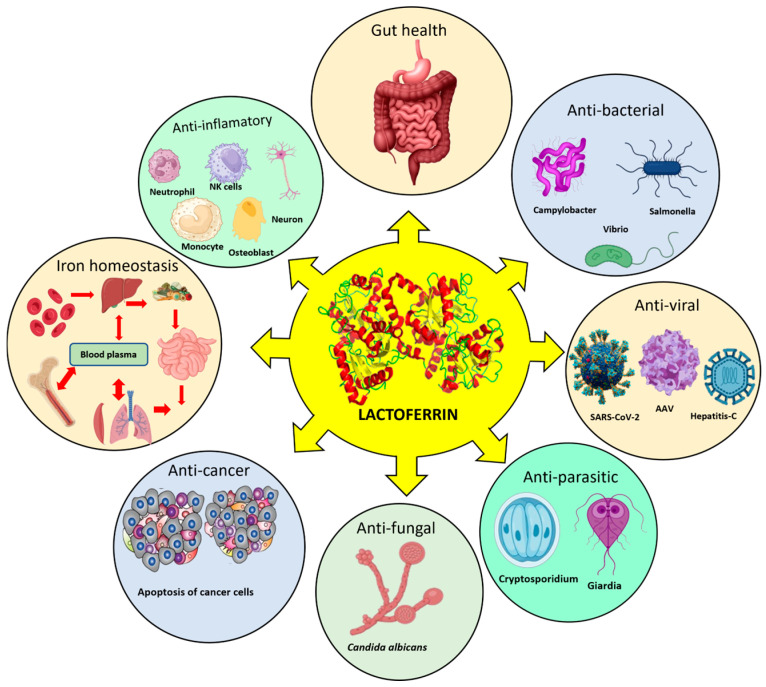
Biological functions of the lactoferrin protein. This protein exhibits anti-inflammatory, antibacterial, antiviral, antiparasitic, antifungal, good gut health, iron homeostasis, and anticancer properties.

LF is often referred to as “pink gold” due to its distinctive salmon-pink color, a result of its iron content [2,8]. This iron-binding property enhances iron absorption in both breastfed infants [8,9] and adults; therefore, LF can be utilized as a functional ingredient in addressing the global challenge of iron deficiency [10]. LF plays several crucial physiological roles, including antimicrobial properties, maintenance of gastrointestinal health, regulation of inflammatory processes, and support the immune system. These functions make LF nearly indispensable for health and growth across all age groups [11] (Figure 2).

## 2. Therapeutic Properties of Lactoferrin

### 2.1. Antipathogenic Properties

LF plays a crucial role in the body’s defense mechanism against infectious agents due to its ability to bind and sequester iron, thereby inhibiting microbial growth. It directly targets bacteria [12,13], viruses [14,15], and fungi [16], reducing their infectivity to host cells. Additionally, LF contributes to iron metabolism and transport by ensuring iron is available for physiological needs while minimizing excess levels that could support pathogen growth.

### 2.2. Anti-Inflammatory Effects

LF has been shown to modulate immunological responses and exhibit anti-inflammatory properties within the body [17]. It plays a crucial role in regulating excessive inflammation and tissue repair processes [18,19].

### 2.3. Gut Health

LF inhibits the growth of harmful bacteria, such as *Helicobacter pylori*, in the gut while promoting beneficial intestinal probiotic flora, including *Lactobacillus acidophilus* and *Bifidobacterium bifidum* [20,21]. Additionally, it supports uterine health and contributes to a healthy digestive system [22].

### 2.4. Antiviral Activity

LF exhibits an in vitro inhibitory effect against a number of viruses, including rotavirus (RV), human papillomavirus (HPV), cytomegalovirus (CMV), respiratory syncytial virus (RSV), hantavirus (HV), poliovirus, herpes simplex virus-1 (HSV-1), herpes simplex virus-2 (HSV-2), hepatitis B virus (HBV), hepatitis C virus (HCV), and SARS-CoV-2 [23,24].

### 2.5. Anticancer Activities

LF has demonstrated significant toxicity against a broad spectrum of cancer cells by inducing cell cycle arrest and causing cytoskeletal damage, ultimately leading to apoptosis of the targeted malignant population. Furthermore, LF reduces cell migration by regulating proteins associated with the cell cycle [25].

## 3. Commercialization of Lactoferrin as a Food Supplement

LF is typically categorized as Generally Recognized as Safe (GRAS) by the United States Food and Drug Administration (FDA). Moreover, the U.S. Department of Agriculture has approved the use of activated LF to be sprayed on the animal carcasses to reduce bacterial contamination during processing. LF is then applied to the surface that will eventually be packaged, helping to prevent bacterial growth and extend shelf life [26]. Given its various functions, LF can also be utilized as an exogenous dietary additive in the food processing sector. The global market for bovine lactoferrin (bLF) is projected to grow at a compound annual growth rate (CAGR) of 15.8% from 2023 to 2033, increasing from approximately USD 772.39 million to USD 3349.05 million [27]. Numerous multinational companies have begun marketing LF as a food supplement under various trade names (Table 1).

## 4. Conventional Lactoferrin Purification Methods

Several techniques have been developed to purify LF from dairy products, emphasizing its properties, including metal ion affinity, molecular weight, and solubility.

These characteristics play a crucial role in the purification process, enabling effective isolation and refinement.

### 4.1. Fractionation and Precipitation

The extraction of LF involves several key steps. Milk products containing LF are first subjected to centrifugation or filtration to eliminate larger particles. Next, LF is selectively extracted using ammonium sulfate and ethanol precipitation. According to Luo et al. [28] 48% of bLF was extracted from milk using 80% (NH_4_)_2_SO_4_ by ammonium sulphate precipitation, followed by precipitation with 60% ethanol. However, the yield of purified LF was lower compared to other purification methods [28].

### 4.2. Ion Exchange Chromatography

At near-neutral pH values, LF carries a positive charge due to the presence of the amino acids arginine (R) and lysine (K), making it suitable for separation via ion exchange chromatography using diethylaminoethyl cellulose (DEAE-C) or carboxymethyl Sepharose (CM Sepharose). It was observed that cationic exchange chromatography resulted in a recovery rate of nearly 90%. However, the use of excessive pressure and lengthy processing time renders this method is cost-ineffective for large-scale production [29].

### 4.3. Size Exclusion and Affinity Chromatography

Size exclusion chromatography separates molecules based on their size. Affinity chromatography takes advantage of LF’s specific binding to antibodies and ligands. Immunoaffinity chromatography reported a yield of 77% with more than 90% purity from sweet whey [30].

### 4.4. Ultrafiltration and Dialysis

This method facilitates the purification and further concentration of LF by removing low-molecular-weight molecules and salts. Ultrafiltration utilizes membranes with specific pore sizes to separate LF from smaller molecules, while dialysis employs buffer exchange to eliminate undesirable salts and small molecules. The degree of purification achieved for human lactoferrin (hLF) exceeded 95% using ultrafiltration and dialysis techniques [31].

### 4.5. High-Performance Liquid Chromatography (HPLC)

HPLC is an advanced chromatographic method used to achieve very high LF purity. Reverse-phase HPLC operates based on hydrophobic interactions. A successful separation of LF from a mixture of proteins was accomplished using this technique [32]. In 2020, Pang Jing and his colleagues integrated HPLC with immunoaffinity magnetic purification (HPLC-FL) for the purification of bovine lactoferrin (bLF) from dairy products, which enabled calibration over a range of 0.8 to 30 μg/mL [33].

Traditional methods of extracting proteins from milk often result in low yields and high production costs. In recent decades, the use of various expression systems, such as bacteria, yeast, animals, and plants, has opened new avenues for the expression of recombinant proteins, helping to address the challenges posed by conventional LF purification systems.

## 5. Recombinant Lactoferrin Biopharming Systems

**Figure 3:**
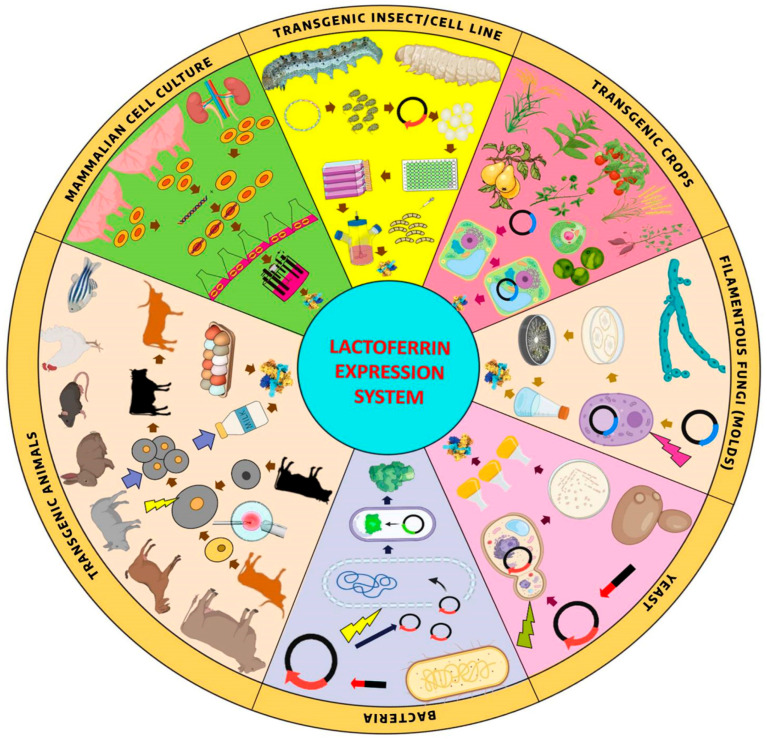
Approaches for producing recombinant lactoferrin using various expression systems.

“Biopharming” refers to the use of genetically modified organisms to produce therapeutic proteins and valuable compounds. The Biopharming commonly known as “molecular farming”, this approach has gained popularity due to its ability to generate specific proteins in controlled environments, resulting in quality and purity of LF that far surpasses traditional dairy sources. The utilization of bacterial and yeast systems minimizes the risk of contamination and facilitates bulk production to meet the increasing demand of LF (Figure 3).

Several expression systems are currently available for the production of pure LF from various sources, including bacteria (*Escherichia coli*), yeast (*Komagataella phaffii*), filamentous fungi (*Aspergillus oryzae*), insect cells (Sf9), and mammalian cells (Chinese Hamster Ovary, CHO) [34]. Animal bioreactors, which include transgenic cattle, goats, swine, rabbits, mice, chickens, and fish, present a promising method for producing LF in their biological fluids, such as milk, eggs, and meat, by utilizing various biotechnological approaches. Additionally, transgenic crops can be engineered to produce varying levels of LF, thereby reducing the reliance on animal bioreactors.

### 5.1. Prokaryotic Expression Systems

The prokaryotic expression system is highly optimized, user-friendly, and scalable, featuring a very short production timeline. Numerous recombinant proteins have been successfully expressed using the *Escherichia coli* expression system [35] (Table 2).

Numerous studies have demonstrated the efficacy of various LF variants produced by *E. coli.* For example, buffalo N-lobe LF was expressed using *E. coli* BL21(DE3) strain with the pQE30 vector, achieving a concentration of approximately 1 mg/mL [36]. In contrast, the expression of hLF utilized the pET28a+ vector, resulting in significantly higher concentrations, around 2.9 mg/mL [37]. Additionally, the hybrid peptide LF15-CA8, composed of bovine lactoferricin (LfcinB) and Cecropin A (CA8), was produced efficiently using the pGEX-4T-2 vector, with concentrations ranging from 5.1 to 10 mg/L [38]. Further successful products included bovine lactoferrin (bLF), which reached concentrations of up to 15.3 mg/L [39], and full-length mouse lactoferrin (mLF), yielding 17 mg/L [40] when expressed with the pET32a vector.

Other bacteria are also of interest for the large-scale production of LF; *Rhodococcus erythropolis* has been utilized for the expression of the C-lobe of bLF using the PTipLCH1.2 vector, achieving a production titer of 3.6 mg/mL [42]. Next in line is *Bacillus subtilis*, which, by employing the Pveg promoter system, was able to express bLF at concentration of 7.5 mg/L [47] and 29.6 mg/L [48], respectively. Additionally, *Bacillus* strains P245 and P263 produced six repeats of lactoferricin (6Lfcin), demonstrating good antibacterial properties when using the pPtrnQ-6LFB-GFP vector [46].

*Lactococcus lactis* and *Lactobacillus plantarum* have also been utilized for LF production. Using the vector pAMJ1653, a camelid lactoferrin chimeric peptide was expressed at a concentration of 0.13 mg/mL in the *Lactococcus lactis* P170 strain [49]. Meanwhile, Lactobacillus plantarum produced porcine lactoferrin (pLF) at a rate of 8.8 mg/L within 36 h [50] and achieved a concentration of 27.2 μg/mL using the pPG612.1 vector [51].

Genetically modified bacterial systems used for the production of LF face significant challenges. They often lack essential post-translational modifications, such as glycosylation. Additionally, these systems may occasionally produce inclusion bodies [53].

### 5.2. Eukaryotic Expression Systems

#### 5.2.1. Yeast

Yeasts such as *Komagataella phaffii* (formerly known as *Pichia pastoris*), *Pichia methanolica*, and *Saccharomyces cerevisiae* can glycosylate proteins without producing endotoxins, making this group well-suited and economically viable for protein expression in standard laboratory conditions. The *K. phaffii* can express and secrete heterologous proteins during high-density fermentation, enabling the synthesis of diverse array of proteins (Table 3).

Recent studies have made significant advancements in the production of LF in *P. pastoris.* In 2024, Yen and colleagues [54] expressed pLF in *P. pastoris* GS115, achieving an impressive expression level of approximately 2.8 g/L, a substantial increase from the previously reported 87 mg/L. This recombinant pLF also demonstrates antimicrobial properties against a variety of pathogens and cancer cells. In another study conducted in 2024, Lv and his team expressed lactoferricin B (LfcinB) at 11.5 mg/L in *P. pastoris* GS115, which they, further enhanced to 28.8 mg/L by utilizing a hybrid signal peptide [55]. Additionally, Wang and colleagues expressed hLF in *K. phaffii*, achieving intracellular levels of 137.6 mg/L and extracellular levels of 304.6 mg/L in shake flasks, with a subsequent scale-up to 1.7 g/L in a 3 L bioreactor [56]. In 2024, Zhang et al. cloned LF genes into the pPIC9K vector in *P. pastoris* GS115, reporting a yield of 824.93 mg/L of bovine lactoferrin (bLF), with antimicrobial activities [57]. These developments indicate the commercial viability of LF production, particularly in relation to Helania Inc.’s Effera™, New York, NY, USA with over 98% purity [58]. Furthermore, Elnaz et al. [63] expressed Arabian camel lactoferrin (cLF) in *P. pastoris* (PichiaPink™) in 2016, demonstrating notable antibacterial effects against *S. aureus* [63].

In 2013, Xi et al. demonstrated the expression of the tri-hybrid peptide LHP7 in *P. pastoris* strain X-33 [66]. They achieved a yield of 0.906 g/L of the peptide after 108 h of methanol induction, which exhibited antimicrobial activity against *Streptococcus pneumoniae* and *S. aureus*. The fusion peptide LFA-LFC was produced by Tang et al. in 2012 at a fermentation level of 0.27 ± 0.12 mg/L in *P. pastoris* (KM71) [67]. In 2008, Choi et al. [71] reported a yield of 99.8 mg/L for recombinant hLF when expressed in *P. pastoris* (yAS309). They measured the production level at 0.1 mg/mL, which inhibited *E. coli* from piglets, but showed no effect against the standard *E. coli* ATCC 25922 strain. Additionally, in another study, pLF was successfully produced in *P. pastoris* (GS115) using the pPICZαC vector with the AOX1 promoter, achieving a yield 760 mg/L of pLF in cytoplasm [75].

*P. pastoris* (KM71) was recently used to express hLF at a rate of 115 mg/L using the pPIC3.5K vector with the AOX1 promoter [76]. LF expression in *P. pastoris* (SMD 1168) has also been reported by Wang and colleagues, who employed the glyceraldehyde 3-phosphate dehydrogenase (GAPDH) promoter in this system; however, without a signal sequence, the protein aggregated in the cytoplasm. Optimization of pH and the supplementation of ferric ions enhance the LF expression [77]. In their analysis of LF production, Paramasivam et al. (2002) expressed a functional recombinant equine lactoferrin (eLF) using the pPIC9K vector in *P. pastoris* GS115, yielding 40 mg/L [78]. The purified protein exhibited a strong affinity for iron ions, demonstrating its iron-binding activity. The potential of *Pichia methanolica* for expression has also been evaluated. A significant challenge in its use for protein production is its high rate of unsatisfactory translocation and N-glycosylation, which differs from human glycosylation and may lead to potential immunogenic effects. Despite these challenges, *P. pastoris* remains the preferred eukaryotic expression system for LF production. Similarly, filamentous fungi are also employed for recombinant protein production.

#### 5.2.2. Filamentous Fungi (Molds)

The production of LF in filamentous fungi offers several advantages, including high yield, proper protein folding, and glycosylation patterns that closely resemble those found in mammals [83]. Various species of *Aspergillus* have shown promising results in LF production, achieving significant yields through the use of different vectors and promoters (Table 4). For example, 25 mg/L of hLF was produced in *Aspergillus oryzae* using the pAhLFG vector driven by the α-amylase promoter [18,84], and in *Aspergillus nidulans* using the pGEM4 vector under the control of the alcohol dehydrogenase promoter (alcA) [18]. Additionally, *A. awamori* produced 2 g/L of hLF using the pPLF-19 vector with the glucoamylase promoter (GAP) [85,86], while 12 mg/L of murine LF was generated in *A. awamori* using the p26mLF vector with the SP6 promoter [87]. Furthermore, *A. nidulans* produced 5 µg/mL of hLF using the pGEM4 vector with the alcA promoter [88]. However, despite these encouraging results, challenges persist, particularly due to the antimicrobial properties of LF, which can inhibit the growth of host microorganisms and restrict cell density during fermentation.

#### 5.2.3. Transgenic Insect and Insect Cell Line Expression Systems

Transgenic insects and insect cell lines offer significant advantages for the production of LF, including high yields of properly folded proteins with essential post-translational modifications. The Baculovirus Expression Vector System (BEVS) enhances both yield and protein solubility, making it ideal for industrial use. Silkworms and BEVS have been employed for LF production (Table 5), achieving remarkable yields through various vectors and promoters. For example, 12.07 mg/g of hLF was produced in silkworm strain 34 [89], while *B. mori* ovary cells yielded 13.5 µg of hLF per 1–2 × 10^5^ cells [90]. Additionally, 205 µg of pLF was obtained per pupa in *B. mori* cells [91], and 65 µg of hLF was produced per mL of hemolymph using the *B. mori* Nuclear Polyhedrosis Baculoviral expression system [92]. In *Spodoptera frugiperda* (Sf9) cells, 10 mg of bLF (N lobe) was achieved per mL of culture [93], while 9.5 mg/L of hLF was produced using the p8hLFc vector and nuclear polyhedrosis system [94]. Furthermore, 10–15 mg/L of hLF was obtained with the VL1392 vector [95]. However, the antimicrobial properties of LF pose a challenge, as they can inhibit host cell growth and hinder the attainment of the necessary cell densities.

#### 5.2.4. Mammalian Cell Culture System

Mammalian cell culture systems provide a significant advantage in the production of complex proteins that possess human-like post-translational modifications, which are essential for therapeutic applications. (Table 6). hLF has been expressed in bovine mammary epithelial cells (BMECs) using the PiggyBac transposon system in conjunction with the bovine β-casein promoter. This study highlights the versatility of transposon technology in generating human proteins within important domestic animal species [96]. Furthermore, human urine-derived stem cells (USCs) have emerged as a promising alternative for LF expression, offering a non-invasive method for protein production [97]. In contrast, bLF has yielded only 6 µg/mL in BMECs [98], underscoring the importance of these cells in producing species-specific proteins that can enhance immune response.

In 2017, Yuan et al. [99] demonstrated TALEN (Transcription Activator-Like Effector Nuclease)-mediated gene editing in caprine fetal fibroblast (CFFB) cells by replacing the goat beta-lactoglobulin gene with the *hLF* gene. Chinese hamster ovary (CHO) cells can produce hLF at concentrations exceeding 200 mg/L using the pTT5 vector [100]. These cells are well-established in biopharmaceutical production due to their defined growth conditions. hLF was expressed in goat mammary gland epithelial cells (GMECs) and mouse mammary epithelial cells (C127 cells) using the pBC1-hLF-Neo vector, which incorporates the goat beta-casein gene promoter [101]. Bovine mammary epithelial cells (BMECs) expressed hLF with the pMD 18-T and pEGFP-C1 vectors utilizing the cytomegalovirus (CMV) promoter, achieving a yield of 1135.8 ± 534.3 µg/mL [102]. Human embryonic kidney (HEK-293) cells produced a similar yield using the pShuttle vector with the CMV promoter [103].

Other notable achievements include the utilization of the pBL1 vector in rat mammary epithelial cells [104] and the application of the pαS1 vector in the HEK-293 cell line, which yielded up to 0.6 g/mL [105]. Mouse mammary epithelial (HC11) cells achieved production levels of 150–200 µg/mL with the pBL1 vector [106], while baby hamster kidney (BHK) cells produced 20 mg/L using the pNUT vector in conjunction with the metallothionein promoter [107]. The production of LF in mammalian cell culture expression systems is associated with high costs and complexity, necessitating specialized growth media, specific culture conditions, and extensive handling, which ultimately increases production costs compared to other expression systems.

#### 5.2.5. Animal Bioreactors

Animal bioreactors are increasingly being utilized for the production of recombinant proteins in their biological fluids, such as milk, eggs, and meat. Employing animals as bioreactors is a cost-effective approach that facilitates the incorporation of appropriate post-translational modifications in the proteins produced.

##### Transgenic Cattle

Transgenic cattle represent an effective means of producing hLF, as they can generate substantial quantities in their milk, making extraction cost-effective. Recent studies have enhanced lactoferrin expression through various techniques (Table 7 and Table 8), including the use of bacterial artificial chromosome (BAC) vector combined with somatic cell nuclear transfer (SCNT) [108], which achieved yields ranging from 4.5 to 13.6 g/L of hLF. Another method involved the use of pIRES2-EGFP vector, resulting in yields of 0.0098 to 0.011 mg/mL of hLF [109]. Additionally, BAC vector and the β-casein promoter were utilized through microinjection, yielding 2.5 to 3.4 g/L of hLF [110]. Furthermore, microinjection of bovine αS1 casein has been employed to produce hLF, achieving concentrations of 1.5 to 2.0 g/L in milk [111,112]. However, this method raises ethical concerns regarding genetic modification and the potential unforeseen consequences of genetic alterations or environmental influences.

##### Transgenic Goats

Transgenic goats present a promising approach for producing hLF due to their ability to generate substantial quantities in their milk, which includes human-like post-translational modifications. This characteristic makes them a more efficient alternative compared to transgenic cattle.

Recent advancements in the production of hLF in transgenic goats have employed a variety of vectors and methods (Table 7). The pBC1 vector, which features the goat β-casein promoter, produced 2.60 g/L of hLF in milk through microinjection [113]. In 2019, Semak et al. [114] improvised this technique, achieving 16 g/L by microinjecting into the zygotic male pronucleus. The pCL25 vector, utilizing a goat β-casein-CMV chimeric promoter, yielded 4.7 mg/mL of hLF by the fourth day of lactation through microinjection into goat fetal fibroblasts (GFBs) [115]. In 2017, Yuan et al. [99] employed a TALEN-mediated strategy with the pBLC-TK vector to create biallelic knock-in fibroblasts. Zhu et al. [116] achieved approximately 1.6 g/L using the pIRES2-EGFP vector via microinjection in 2016. Research by Meng et al. [119] in 2013 and Zhang et al. [117] in 2015 demonstrated varying expression levels with the pBC1 vector [117,119]. An Li You et al. [120] reported a yield of 2.1 g/L with the pBLC14 vector through SCNT in 2012. The versatility of the pBC1 vector was further illustrated by Yu et al. [121] in 2012a, who achieved up to 30 g/L through microinjection. In 2012, Goldman et al. [122] recorded 10 g/L from the pBC1 vector via microinjection, while Wan et al. [123] in 2012 successfully produced transgenic kids using SCNT and liposome transfection. Meng et al. [101] achieved LF expression in culture medium through lipofection with pBC1-hLF-Neo in 2011. Similarly, Zhang et al. [124] reported 0.765 mg/mL using the pBC1 vector in 2008. In 2008, Li et al. [125] cloned embryos utilizing the pGBC2LF vector, which features the goat β-casein promoter [125]. Han et al. [126] reported a yield of 2 g/L using an adenovirus vector (pAd-hLF) in 2007, while Cui et al. [118] achieved 1.3 g/L with a BAC (pBHC) through TALEN-induced recombination in 2015 [117]. Lastly, Yu et al. [121] in 2012 demonstrated the adaptability of the pBC1 vector, reaching an impressive 30 mg/mL of hLF in milk via SCNT [121]. However, genetic modification poses potential risks to the health of cattle and goats and raises ethical concerns, necessitating careful consideration for responsible development and use.

##### Transgenic Swine

Transgenic pigs have been utilized to express LF through SCNT and gene editing techniques such as CRISPR/Cas9 (Table 8). In 2020, Han et al. [127] employed SCNT and CRISPR/Cas9 to achieve a site-specific knock-in of the LF gene at the *CSN1S1* locus, ensuring consistent production of pLF. Additionally, hLF was expressed using a pBAC vector with a bovine β-casein promoter via SCNT, which yielded a concentration of 6.5 g/L of LF in milk [128].

##### Transgenic Rabbits

Transgenic rabbits have been employed as bioreactors for LF production. Their short gestation periods facilitate rapid production cycles, and their manageable size makes them ideal for recombinant protein synthesis. Various methods have been explored for producing LF in transgenic rabbits, each yielding different results (Table 8). Firstly, the pShuttle-CMV vector paired with the CMV promoter enabled recombinant adenovirus-mediated gene transfer, achieving a yield of 2.3 mg/mL of LF in milk [129]. The pEGFP-N1 vector, utilizing an enhanced cytomegalovirus (eCMV) promoter, produced approximately 103 ± 20 µg/L through sperm-mediated gene transfer (SMGT) [130]. Additionally, using the pCMV vector, a yield of 2.3 mg/mL of LF was also achieved [131]. However, ethical concerns regarding genetic modification and animal welfare, along with regulatory challenges and the resource-intensive nature of these methods, complicate the path to commercialization.

##### Transgenic Mice

Transgenic mice serve as effective bioreactors due to their relatively short gestation periods, which facilitate the rapid production and screening of generations. This efficiency aids in the identification of successful transgenic lines for LF production. Several approaches have been explored for producing LF in transgenic mice (Table 8). For example, the pBC1 vector paired with a goat β-casein promoter achieved remarkable expression levels ranging from 15.3 to 160 g/L of hLF in milk [122]. Another strategy utilized a hybrid multiplex promoter with a cytomegalovirus (CMV) enhancer, resulting in LF expression levels between 1.17 and 8.10 mg/mL [133]. In SCNT experiments using the BAC vector (pBAC-hLF-hLZ-Neo), researchers reported LF yields of 0.21 to 1.76 g/L [134]. A noteworthy method involved the use of the pBC1 vector with a beta-casein gene promoter, which successfully expressed LF in mouse mammary epithelial cells (C127) and in the culture supernatant [101]. Furthermore, the T-protruding pCR3 vector with a bovine α-lactoalbumin promoter achieved an expression level of 120 × 13.6 mg/L of pLF through microinjection [135]. However, one drawback of utilizing transgenic mice for LF production is the variability in expression levels, which can be influenced by factors such as the insertion site of the transgene. This variability may lead to inconsistent yields and functionality.

##### Transgenic Chickens

Transgenic chickens offer unique advantages for the production of LF, particularly by utilizing eggs as bioreactors, which facilitates efficient and large-scale production. This approach can significantly lower costs compared to traditional biopharmaceutical production techniques. A pertinent study demonstrated the effectiveness of this method by employing the pBluescript II KS (+) plasmid vector in conjunction with the human CMV promoter. This combination enabled the production of hLF via a recombinant chicken embryo lethal orphan (CELO) adenovirus in chicken embryos, resulting in concentrations ranging from 0.1 to 0.3 mg/mL in the embryo culture medium [146].

##### Transgenic Fish

Transgenic fish offer significant advantages for the production of LF and the enhancement of meat quality, including accelerated growth rates, increased yields, and improved nutritional profiles. Additionally, these fish can be engineered for disease resistance, which helps reduce the need for antibiotics. Various research methodologies have been employed to generate LF in fish models (Table 7). For instance, in *Danio rerio* (zebrafish), researchers utilized the pCS2+ vector in conjunction with the simian cytomegalovirus (sCMV IE94) promoter for microinjection, achieving a hLF expression level of 64 ng/mL during the fry stage [148]. In 2010, Lin et al. [147] successfully expressed bovine lactoferricin protein by employing the pZBGFP vector with a beta-actin promoter through microinjection in fish embryos. In *Ctenopharyngodon idellus* (Chinese grass carp), the pCAgcGH vector, which utilizes the common carp β-actin promoter, facilitated SMGT via electroporation, resulting in transgenic fish that were resistant to the grass carp hemorrhage virus (GCHV) [149]. These studies highlight the potential of transgenic fish not only for LF production but also for improving meat quality and enhancing disease resistance.

#### 5.2.6. Transgenic Crops

Transgenic crops that express LF may significantly enhance their nutritional value and boost immunity, making them a cost-effective source of LF. Furthermore, these crops could reduce reliance on LF derived from animal sources and contribute to more sustainable food production with a decreased environmental impact.

##### Transgenic Rice

Transgenic rice offers both advantages and disadvantages in the production of LF. It can yield LF in amounts ranging from micrograms per gram to several percent of total soluble protein, making it a viable option for large-scale production while enhancing the nutritional value of this staple food. Research on LF expressions in *Oryza sativa* has explored various vectors and promoters (Table 9). Lee et al. [150] utilized the pCAMBIA-1300 vector, which is regulated by the rice actin promoter, achieving a purity range of 0.12% to 100% and producing 987 μg of recombinant protein per 815 mg of total extractable protein in 2020. In 2017, Funakoshi et al. [151] employed the binary plasmids pIG260 and pIG261, driven by the maize ubiquitin-1 promoter, to produce LF, which demonstrated strong antimicrobial properties.

Other studies have successfully reported LF expression in rice, including Lee et al. (2010) and Lin et al. (2010), who employed the pCAMBIA1300 vector with the cauliflower mosaic virus 35S promoter (CaMV 35S), achieving 0.1% and 0.45% of rice bran weight and total dry weight, respectively [152,153]. Additionally, Rachmavathi et al. [154] in 2004 and Takase et al. [155] in 2005 utilized binary vectors with the maize ubiquitin-1 promoter and the pCAMBIA 1301 vector with the CMV 35S promoter, achieving 2.0 mg/g and 2.1 mg/g of dehusked seeds, respectively. Furthermore, Suzuki et al. [156] in 2003 and Nandi et al. [157] in 2002 employed the pAPI137 and pAPI135 vectors with the rice actin promoter, achieving yields of 2–4% and 0.5–5.0 g/kg of dehusked rice, respectively.

##### Transgenic Tobacco

*Nicotiana tabacum* and *Nicotiana benthamiana* offer significant advantages for LF production, achieving yields ranging from 0.1 to 1.8% of total soluble protein, along with a short growth cycle that facilitates rapid scalability. Research has demonstrated effective production techniques, such as those reported by Miura et al. [13] in 2023, who achieved a yield of 40 µg/g of fresh mass of hLF using the pTKB3 vector with the CaMV 35S promoter in *N. benthamiana*. In *N. tabacum*, the pART27 vector employing the CaMV 35S system resulted in camel lactoferrin (cLF) expression at 1.5% of the total soluble protein [158]. Additionally, *N. tabacum var. Xanthi* expressing bLF through the pCAMBIA 1301 vector with the CaMV 35S promoter yielded 0.5% of total soluble protein [159]. These findings underscore the versatility and efficacy of various vector systems in producing valuable proteins within plant systems.

##### Transgenic Potatoes

*Solanum tuberosum* is a plant used for evaluating host productions of LF. Buziashvili et al. [166] in 2020 were able to express hLF in their construct by using the CaMV 35S promoter on potato to yield total soluble proteins of 0.05%. Chong et al. [167] in 2000 exploited the mas P2 potato promoter to express hLF, yielding values anywhere between 0.01 to 0.1% of total soluble proteins.

##### Transgenic Tomato

The tomato (*Lycopersicon esculentum*) has recently been utilized as a research model to evaluate its productivity of LF. Buziashvili et al. [168] demonstrated in 2020 that the expression of hLF reached levels of 0.5% of total soluble protein after the introduction of the pBI121 vector into *L. esculentum* cells, under the control of the CaMV 35S promoter. In a separate study conducted by Lee et al. [169] in 2002, a similar approach using the same vector and promoter resulted in hLF expression at a lower level of 0.1% of total soluble protein [169].

##### Other Transgenic Crops

In a series of studies involving various plant species, researchers expressed human and bovine LF using different vectors and promoters. Malnoy et al. [170] in 2003 reported that pear (*Pyrus* sp.) transformed with the pBI121 vector under the CaMV 35S promoter achieved bLF expression at 0.3% of the total soluble protein.

In the context of *Panax ginseng* and *Acanthopanax senticosus*, Jo and Kwon et al. [171] in 2006 found that the latter could express hLF at 3.6% of total soluble protein using the PCAMBIA2300 vector with an oxidative stress-inducible peroxidase (SWPA2) promoter. In contrast, Kwon et al. [172] reported a 3% expression level in a Korean ginseng cell line. For *Triticum aestivum* (wheat), Han et al. [173] in 2012 measured bLF levels ranging from 21 to 67 ng/mg of tissue using the pAM4424 vector with the CaMV 35S promoter. These studies underscore the versatility and effectiveness of various expression systems in different plant species for producing LF with a range of antimicrobial properties.

Different expression systems produce varying amounts of LF, each with its own advantages and limitations (Table 10). Yeast systems are commonly used because they allow for high-density fermentation and easy genetic modifications, with LF concentrations ranging from 0.00027 to 3.5 mg/mL. However, a key drawback is their lack of human-like glycosylation, along with the potential for protein misfolding, which can affect the functionality of LF for pharmaceutical applications. On the other hand, transgenic animals, particularly goats, can produce much higher levels of LF, ranging from 0.765 to 30 mg/mL in their milk. Goats are preferred for large-scale production because they produce more milk and have more reliable glycosylation, making them more cost-effective for industrial production. Mice, while useful for small-scale production and genetic studies, produce lower amounts of LF and have inconsistent expression levels, making them less efficient for large-scale production.

## 6. Future Prospects

To meet the growing demand for LF, it is essential to optimize production systems. Current strategies focus on enhancing biopharming platforms, including microbial fermentation, transgenic plants, and animal bioreactors, to improve yields, reduce production costs, and ensure product quality [180]. One effective approach involves adjusting codon usage to better align with the translational machinery of the host of the organism’s, which significantly boosts protein expression [178]. This codon optimization enhances mRNA translation efficiency and folding, ultimately resulting in higher yields of functional LF. Additionally, developing strong, inducible chimeric promoters tailored to specific expression systems [49,115,142], such as those in bacterial or plant hosts, can enhance transcriptional activity and ensure high-level expression of LF.

Further improvements can be achieved by engineering host cells to enhance accurate protein folding and essential post-translational modifications like glycosylation [181]. These modifications are vital for maintaining LF’s biological activity and functional properties. Additionally, optimizing signal peptides [182] for more efficient secretion of LF into the extracellular environment helps minimize the need for complex and costly purification steps, thereby improving both yield and purity. Another effective strategy involves the co-expression of molecular chaperones within expression systems [183], which facilitates proper protein folding, reduces aggregation, and increases the overall yield of biologically active LF.

Synthetic biology tools provide valuable opportunities by facilitating the design of dynamic genetic circuits, such as feedback loops and adjustable promoters [184] that enhance LF production under varying growth conditions. Additionally, optimizing ribosome binding sites within mRNA sequences can further improve translation initiation [77,115,133], particularly in bacterial and yeast systems, resulting in increased protein output.

Concurrently, innovations in bioprocess engineering, particularly in metabolic engineering and process optimization, play a crucial role in enhancing production performance. Refining fermentation parameters and incorporating high-throughput screening, along with real-time monitoring methods, are essential for achieving optimal growth conditions and maximizing LF yields [185]. Developing novel host strains, such as metabolically engineered bacteria, yeast, or plant lines with improved stress tolerance and biosynthetic capabilities, can significantly increase production efficiency, lower costs, and enhance scalability [186]. Modifying the ubiquitin–proteasome pathway in host cells to minimize the premature degradation of proteins [187] can help stabilize LF, allowing for it to remain functional throughout the expression and purification processes.

Integrating various genome editing technologies, including CRISPR [127], Zinc Finger Nucleases (ZFN), and Transcription Activator-Like Effector Nucleases (TALEN) [99,118], is essential for ensuring stable and consistent expression of LF across different production platforms. Furthermore, ongoing research into the therapeutic potential of LF is crucial for expanding its market applications, particularly in the fields of functional foods and dietary supplements [188,189].

Recent innovations in plant-based and yeast expression systems have increasingly focused on replicating human-like glycosylation to enhance the functional properties of recombinant therapeutic proteins such as LF. Researchers have successfully engineered *Arabidopsis* and tobacco plants to express host glycosylation enzymes, thereby enabling these hosts to produce glycoforms that more closely mimic those found in human proteins [190]. Similarly, advances in genome editing particularly through the application of CRISPR-Cas9 showed precise modification of glycosylation pathways in mammalian systems such as CHO cells. The integration of human glycosylation genes into these hosts has resulted in the production of LF with glycan profiles that closely resemble its native human form [191,192].

In parallel, artificial intelligence (AI) model-guided metabolic engineering strategies have facilitated significant advancements in microbial expression systems. Engineered bacterial strains, including *E. coli*, have been developed to introduce basic glycosylation pathways, allowing for the incorporation of simple N-linked glycans into proteins such as LF [193]. Although these systems currently lack the complexity of mammalian glycosylation, they offer promising potential for large-scale, cost-effective production. Furthermore, cell-free protein synthesis (CFPS) platforms utilizing purified cellular components in a controlled in vitro environment have emerged as a versatile alternative. These systems allow for precise control over post-translational modifications, including glycosylation, and offer considerable flexibility for the scalable production of functionally active LF [194].

## 7. Conclusions

Biopharming of LF is an exciting field with significant potential for therapeutic applications. Recent advancements in recombinant protein expression systems and bioprocessing techniques indicate a shift towards more efficient and cost-effective production methods. Furthermore, addressing existing knowledge gaps through targeted research will be essential for maximizing the benefits of LF biopharming. Investigating innovative expression systems, optimizing production processes, and integrating with advanced drug delivery technologies will be crucial for the future of LF as a valuable therapeutic protein.

## Figures and Tables

**Table 1:** A list of international players in the lactoferrin market with trade names.

Trade Name	Lactoferrin Type (LF)	Manufacturing Firm, Country
SureStart™ Lactoferrin 7200	Bovine lactoferrin (bLF)	New Zealand Milk Products (NZMP), Auckland, New Zealand
Effera™	Human lactoferrin (hLF)	Helaina Inc., New York, NY, USA
Vitalarmor^®^ Lactoferrin	bLF	Armor Protéines, Saint-Herblain, Loire-Atlantique, France
Bioferrin^®^	bLF	Glanbia Nutritionals, Kilkenny, Ireland
Vivinal^®^ Lactoferrin	bLF	Royal Friesland Campina, Amersfoort, Utrecht, The Netherlands
Synlait lactoferrin	bLF	Synlait Milk Ltd., Dunsandel, Canterbury, New Zealand
Inferrin^®^	bLF	Bega Cheese Limited, Bega, NSW, Australia
PUREnFERRIN™	bLF	Freedom food group, Stanbridge, NSW, Australia
Proferrin^®^	bLF	Ingredia SA, Arras, Hauts-de-France, France
LF+	bLF	Turtle tree, Singapore
Valpalf^®^	bLF	Pharmaguida, Milan, Lombardy, Italy

**Table 2:** List of studies producing recombinant lactoferrin using prokaryotic expression system.

Species (Strain)	Vector/Promoter	Type of Lactoferrin	Yield/Culture	References
*Escherichia coli* BL21(DE3)	pQE30	Buffalo LF N-lobe	01 mg/mL	[36]
	pET28a+	hLF	2.9 mg/mL	[37]
	pGEX-4T-2	Hybrid peptide LF15-CA8 Bovine lactoferricin (LfcinB)-Cecropin A(CA8)	5.1–10 mg/L	[38]
	pET32a	bLF	15.3 mg/L	[39]
	pET28a	Mouse lactoferrin (mLF) (full length)	17 mg/L	[40]
	pET32a	bLF oligomeric peptide-(LfcinB15-W4,10)	74 mg/L	[41]
	pET21d	LfcinB fusion peptide	60 mg/L	[42]
	pGEX-4T-2	LfcinB	02 mg/L	[43]
*E. coli* C43(DE3)	pET32a	Fusion peptide SrtA-LfcinB *Staphylococcus aureus* sortase A (SrtA)-N-terminus of LfcinB	LfcinB-1.32 ± 0.07 mg/500 mL	[44]
	pET21b	pET21b-(fLfcinB-bmIFc2)2 co-expressionbmIFc2	4.1 ± 01 mg/500 mL	
*Rhodococcus erythropolis*	pTipLCH1.2	bLF C-lobe	3.6 mg/mL	[45]
*Bacillus subtilis* P245 and P263	ptrnQ promoter	Six tandem repeats of Lfcin	Antimicrobial activity	[46]
*Bacillus subtilis*	pMA0911/ Pveg promoter	bLF C-lobe	7.5 mg/L	[47]
	pMA0911/ Pveg promoter	bLF N-lobe	29.6 mg/L	[48]
*Lactococcus lactis* (P170)	pAMJ1653 expression vector	Camel lactoferrin (cLF) chimeric peptide (lactoferrampin–lactoferricin)	0.13 mg/mL	[49]
*Lactobacillus plantarum*	pPG-pLF	Porcine lactoferrin (pLF)	8.8 mg/L (36 h)	[50]
*L. plantarum*	pPG612.1 expression vector	pLF	27.2 µg/mL	[51]
*Lactobacillus casei*			20.5 µg/mL	
*Lactobacillus paracasei*			21.6 µg/mL	
*Lactobacillus* *pentosus*			21.6 µg/mL	
*L. casei*	pSD	hLF	10.6 mg/mL	[52]

**Table 3:** List of studies producing recombinant lactoferrin using a yeast expression system.

Species (Strain)	Vector/Promoter	Type of Lactoferrin	Yield/Culture	References
*1. Pichia pastoris* (*Komagataella phaffii*)				
*P. pastoris* (GS115)	pPICZαC/G1 (PG1)	pLF	2.8 g/L	[54]
*P. pastoris* (X-33)	X33-pPICZɑA-PAOX1- Lfcin	LfcinB	19.3 mg/L	[55]
	X33-pPICZɑA-PAOX1-0030-α * Lfcin		28.8 mg/L	
	X33-pPICZɑA-PAOX1-0030-α-PEP1/PEP2 ^#^ Lfcin		150–193 mg/L	
*Komagataella phaffii* (X33)	-	hLF	137.6–304.6 mg/L	[56]
*P. pastoris* (GS115)	pPIC9K/AOX1	bLF	824.93 mg/L	[57]
*Komagataella phaffii* (GS115)	*-*	hLF (Effera™)	>98% purity	[58]
*P*. *pastoris* (GS115)	pPIC9K/AOX1	bLF N-lobe	50.5 mg/L	[59]
	pPIC9K/AOX1	bLF peptide (LfcinB)	Antimicrobial property	[60]
*P. pastoris* (KM71-H)	pJ902/AOX1	bLF	3.5 g/L	[61]
*P. pastoris* (KM71)	PPICZαA/AOX1	Camel lactoferricin (LfcinC)	Antimicrobial property	[62]
*P. pastoris* (PichiaPink^TM^)	pPINKα-HC/AOX1	Arabian LfcinC	Antimicrobial property	[63]
*P. Pastoris* (SMD1168)	pPIC9K/AOX1	hLF- N lobe	458 μg/mL	[64]
*P. pastoris* (GS115)	pPICZaA/AOX1	Ovine LF	>60 mg/L	[65]
*P. pastoris* (X-33)	pPICZA/AOX1	Lfcin tri-hybrid peptide (LHP7)	0.906 g/L	[66]
*P. pastoris* (KM71)	pPICZαA/AOX1	(Fusion peptide) LFA-LFC	0.27 ± 0.12 mg/L	[67]
*P. pastoris* (SMD1168)	pPIC9K/AOX1	hLF	Antimicrobial property	[68]
*P. pastoris* (X-33)	pMD18-T/AOX1	bLF (full length)	88 mg/L	[69]
		bLFA: N-lobe + inter lobe region	485 mg/L	
*P. pastoris* (KM71)	pPIC9K/AOX1	hLF	1200 mg/L	[70]
*P. pastoris* (yAS309)	pPICZA/GAPDH	hLF	99.8 mg/L	[71]
*P. pastoris* (X-33)	pGAPZαC/GAP	Goat lactoferrin (gLF)	2.0 mg/L	[72]
*P. pastoris* (JM109)	pPICZαC/AOX1	Chinese Yak lactoferrin	40 mg/L	[73]
*P. pastoris* (GS115)	pPIC9/AOX1	pLF	0.1 mg/mL	[74]
*P. pastoris* (GS115)	pPICZαC/AOX1	pLF (cytoplasm)	760 mg/L	[75]
*P. pastoris* (KM71)	pPIC 3.5 K/AOX1	hLF	115 mg/L	[76]
*P. pastoris* (SMD 1168)	pGAPZa B/GAPDH	pLF	12 mg/L	[77]
*P. pastoris* (GS115)	pPIC9K/AOX1	Equine LF	40 mg/L	[78]
*2.* *Pichia methanolica*				
*P. methanolica* (PMAD11)	pGEM-3Z/AUG1	pLF N-lobe	Antimicrobial property	[79]
*P. methanolica* (pMAD16)	pMETα A/AUG1	LfcinB	90 mg/L	[80]
*3.* *Saccharomyces cerevisiae (Baker’s yeast)*				
*S. cerevisiae* (BY4741)	pGAL-MF/GAL (Galactose-inducible)	hLF	18.6 mg/L	[81]
*S. cerevisiae* (AB116)	pRL1 vector	hLF	1.5–2 mg/L	[82]

0030-α *: signal peptide; PEP1/PEP2 ^#^: anionic antioxidant peptide; LFA: bovine lactoferrampin; LFC: lactoferricin.

**Table 4:** List of studies producing recombinant lactoferrin using a fungal expression system.

Species (Strain)	Vector/Promoter	Type of Lactoferrin	Yield/Culture	References
*Aspergillus oryzae*	pAhLFG/α-amylase promoter	hLF	25 mg/L	[18]
*Aspergillus nidulans*	pGEM4/alcohol dehydrogenase (alcA)			
*Aspergillus awamori*	pPLF-19/gluco amylase (GAP) promoter	hLF	2 g/L	[85]
*A. awamori*	p26mLF/SP6 promoter	Murine LF	12 mg/L	[87]
*A. awamori*	pPLF-19/gluco amylase (GAP) promoter	hLF	2 g/L	[86]
*A. oryzae*	pAhLFG/α-amylase promoter	hLF	25 mg/L	[84]
*A. nidulans*	pGEM4/alcohol dehydrogenase (alcA)	hLF	5 μg/mL	[88]

**Table 5:** List of studies producing recombinant lactoferrin using transgenic insect and insect cell line expression systems.

Species (Strain)	Vector/Promoter	Type of Lactoferrin	Yield/Culture	References
Silkworm strain 34 (*Bombyx mori*)	piggyBac-based transgenic vector (phSrhLFSer1)/Sericin 1 promoter	hLF expressed in silk glands	12.07 mg/g hLF cocoon shell weight	[89]
Silkworm ovary cell line (*B. mori*)	Recombinant virus generated by co-transfecting pBacPAK-hLf, BacPAK6 vectors to cells.	hLF	13.5 μg/1–2 × 10^5^ cells	[90]
*B. mori*	pBlueBacHisc and HyNPVbaculovirus DNA was co-transfected into Sf-9 cells	pLF	205 μg of rPLF/pupae	[91]
*B. mori*	*B. mori* NPB expression system	hLF	65 μg hLF/mL hemolymph	[92]
*Spodoptera* *frugiperda* (Sf9) cells	VL1392 vector and NPB * expression system	bLF (N lobe)	10 mg bLF N lobe/mL culture	[93]
	p8hLFc vector and NPB * expression system	hLF	9.5 mg/L	[94]
	VL1392 vector and NPB * expression system	hLF	10–15 mg/L	[95]

NPB *: Nuclear Polyhedrosis Baculoviral expression system.

**Table 6:** List of studies producing recombinant lactoferrin protein using a mammalian cell line expression system.

Cell Culture Type	Vector/Promoter	Type of Lactoferrin	Yield/Culture	References
Bovine Mammary Epithelial Cells (BMEC)	PiggyBac transposon + Cre/loxP system/bovine β-casein promoter	hLF	Expressed in culture supernatant	[96]
Human Urine-Derived Stem Cells (HUDSC)	piggyBac transposon	hLF	Higher levels of lactoferrin found	[97]
Bovine Mammary Epithelial Stem Cells (BMESC)	PiggyBac vector	bLF	06 µg/mL	[98]
Goat Fetal Fibroblast Cells (GFFC)	pBLC-TK vector-TALEN-mediated knock in	hLF	(Targeted mutagenesis)	[99]
Chinese Hamster Ovary Cells (CHO)	pTT5 vector	hLF	>200 mg/L	[100]
Goat Mammary Gland Epithelial Cells (GMGEC)	pBC1-hLF-Neo/goat beta-casein gene promoter	hLF	LF expressed in cell culture medium	[101]
HEK293 *	pShuttle-CMV Vector pMD	hLF	1135.8 ± 534.3 µg/mL	[102]
BMEC	18-T, pEGFP-C1/CMV promoter			[103]
Rat Mammary Epithelial Cells (RMEC)	pBL1vector	hLF	hLF detected in culture supernatant	[104]
HEK293	pαS1/bovine αS1 casein promoter	hLF	0.6 µg/mL	[105]
Mouse Mammary Epithelium Cells (HC11)	pBL1vector	hLF	150–200 µg/mL	[106]
BHK Cell culture	pNUT/Metallothionein promoter	hLF	20 mg/L	[107]

* HEK293: human embryo kidney cell culture line 293; BHK: baby hamster kidney cell culture.

**Table 7:** List of studies producing recombinant lactoferrin in transgenic cattle and goats.

Vector/Promoter	Lactoferrin	Transgenesis	Expression Level/Site	References
1. Cattle				
BAC *	hLF	SCNT	4.5–13.6 g/L (milk)	[108]
pIRES2-EGFP/goat β-casein	hLF	SCNT	0.0098–0.011 mg/mL (milk)	[109]
BAC vector/b-casein	hLF	Microinjection	2.5–3.4 g/L (milk)	[110]
Bovine αS1 casein	hLF	Microinjection	1.5–2.0 g/L (milk)	[111]
Bovine αS1 casein	hLF	Microinjection	Successfully expressed	[112]
2. Goat				
pBC1/goat β-casein promoter	hLF	Microinjection	2.60 g/L (milk)	[113]
			16 g/L (milk)	[114]
pCL25/goat β-casein-CMV chimeric promoter	hLF	Microinjection	Avg concentration of 3.89 ± 0.82 mg/mL (milk)	[115]
pBLC-TK vector/TALEN-mediated biallelic knock-in	hLF	Electroporation	(Targeted mutagenesis)	[99]
pIRES2-EGFP/goat β-casein promoter	hLF	Microinjection	1.6 g/L in milk	[116]
pBC1/goat β-casein promoter	hLF	SCNT	Transgenic kids generated	[117]
TALEN mediated knock-in of construct (phosphoglycerol kinase (PGK) promoter-hLF-Neo)	hLF	SCNT	2.3–2.4 mg/mL (milk)	[118]
pBC1/goat β-casein	hLF	SCNT	Transgenic kids generated	[119]
pBLC14/bovine alpha1-casein	hLF	SCNT	2.1 g/L (milk)	[120]
pBC1/goat β-casein promoter	hLF	Microinjection	30 g/L (milk)	[121]
			10 g/L (milk)	[122]
		SCNT and liposome transfection	Transgenic kids generated	[123]
		Microinjection	0.765 mg/mL (milk)	[124]
		Lipofection	LF expressed in cell culture	[101]
pGBC2LF/goat β-casein gene promoter	hLF	SCNT	Cloned embryos developed to blastocyst stage	[125]
Adenovirus expression vector (pAd)/CMV promoter	hLF	Adenovirus mediated transduction	2 g/L (milk)	[126]
pBHC (a bacterial artificial chromosome) /Bovine β-casein promoter	hLF	TALEN-induced homologous recombination	1.3 g/L (milk)	[118]
pBC1 vector/β-casein promoter	hLF	SCNT	30 mg/mL (milk)	[121]

* BAC: bacterial artificial chromosome; SCNT: somatic cell nuclear transfer.

**Table 8:** List of studies producing recombinant lactoferrin in transgenic animals.

Vector/Promoter	Lactoferrin	Transgenesis	Expression Level	References
1. Swine				
CRISPR/Cas9-based site-specific knock-in of LF gene in *CSN1S1* locus	pLF	SCNT	Sustainable LF production	[127]
pBAC/bovine β-casein promoter (Bi-transgenic swine)	hLF	SCNT	6.5 g/L (milk)	[128]
2. Rabbit				
pShuttle-Cytomegalovirus (CMV) vector/CMV promoter (adenovirus-mediated gene transfer into mammary gland)	hLF	Virus-mediated transduction	2.3 mg/mL (milk)	[129]
pEGFP-N1/eCMV promoter	hLF	SMGT *	103 ± 20 µg/L	[130]
pCMV/CMV promoter	hLF	Microinjection	2.3 mg/mL (milk)	[131]
pRB1/rabbit β-casein promoter	hLF	Microinjection	0.2 mg/mL (milk)	[132]
3. Mice				
pBC1/goat β-casein promoter	hLF	Microinjection	15.3–160 g/L (milk)	[122]
Hybrid multiplex promoter/ CMV enhancer	hLF	Microinjection	1.17–8.10 mg/mL (milk)	[133]
	hLF	Microinjection	7–40 ng/mL (milk)	
BAC vector	hLF	SCNT	0.21–1.76 g/L	[134]
pBC1/β -casein gene promoter	hLF	Lipofection	Cell culture medium	[101]
T-protruding pCR3 vector/ bovine α-lactoalbumin promoter	pLF	Microinjection	120 × 13.6 mg/L	[135]
pGEM-3Zf (+)/Bovine β-casein promoter (three-step “gap-repair” strategy)	hLF	Microinjection	16.7 to 29.8 g/L	[136]
pBC1 vector/β-casein promoter	hLF	Microinjection	0.22–40 g/L (milk)	[137]
T-protruding pCR3 vector/bovine R-lactalbumin (RLA) promoter	pLF	Microinjection	10 to 106 µg/mL (milk)	[138]
pBC1 vector	hLF	Microinjection	30 mg/mL (milk)	[139]
BAC vector	hLF	Microinjection	1–8.02 mg/mL (milk)	[140]
pWE cosmid/Bovine β-casein promoter	hLF	Microinjection	1–200 μg/mL	[141]
pBL1vector/chimeric promoter *	hLF	Microinjection	>1 to 200 μg/mL	[142]
pBLC/bovine alpha S1-casein	hLF	Microinjection	0.1 to 36 µg/L (milk)	[143,144]
pBL1vector/chimeric promoter *	hLF	Microinjection	150–200 µg/mL	[145]
4. Chicken				
pBluescript II KS (+) vector/ human cytomegalovirus (CMV) promoter	hLF	Chicken Embryo Lethal Orphan(CELO) adenovirus	0.1 to 0.3 mg/mL (culture medium)	[146]
5. Fish				
*Danio rerio* (Zebra Fish)				
pZBGFP/beta-actin promoter	LfcinB	Microinjection	Expressed protein	[147]
pCS2 (+) vector/sCMV IE94 promoter	hLF	Microinjection	64 ng/mL (fry stage)	[148]
*Ctenopharyngodon idellus (Chinese grass carp)*				
pCAgcGH/common carp β-actin promoter	hLF	SMGT	Transgenic fish resistance to GCHV ^#^	[149]

SMGT: sperm-mediated gene transfer; chimeric promoter *: (exon 1, signal sequence and exon 8, 9 of bovine β-casein gene+ Intron II of rabbit β-globin gene); GCHV ^#^: grass carp hemorrhage virus.

**Table 9:** List of studies producing recombinant lactoferrin in transgenic plants and crop expression systems.

Genus/Species	Vector/Promoter	Type of Lactoferrin	Yield	References
1. *Oryza sativa*				
*Oryza sativa*	pCAMBIA-1300/rice actin	pLF	0.12–100% purity	[150]
*O. sativa L. cv. Nipponbare*	Binary vector (pIG260 and pIG261)/maize ubiquitin-1 promoter	LFcinH	Antimicrobial activity against *B. subtilis* and *E. coli*	[151]
*O. sativa*	pCAMBIA1300/cauliflower mosaic virus 35S promoter (CaMV 35S)	pLF	0.1% of rice bran weight	[152]
*O. sativa*	pCAMBIA1300/CaMV 35S	pLF	0.1% of rice bran weight	[152]
*O. sativa*	pCAMBIA1300/CaMV 35S	hLF	0.45% of total dry weight	[153]
*O. sativa*	Binary vector (pIG200 and pIG211)/maize ubiquitin-1 Promoter	hLF	2.0 mg/g of dehusked seeds	[154]
*O. sativa*	pCAMBIA 1301/CMV35S	hLF-N lobe	2.1 mg/g of dehusked seeds	[155]
*O. sativa* cell culture	pAPI137/rice actin promoter	hLF	2–4% of the total soluble protein	[156]
*O. sativa*	pAPI135/rice actin promoter	hLF	0.5–5.0 g/kg of dehusked rice	[157]
2. *Nicotiana tabacum/Nicotiana benthamiana*				
*Nicotiana benthamiana*	pTKB3/CMV35S	hLF	40 µg/g fresh mass (gFM)	[13]
*Nicotiana tabacum*	pART27/CaMV 35S	Camel LF	1.5% of the total soluble protein (TSP)	[158]
*N. tabacum var Xanthi*	pCAMBIA 1301/CaMV 35S	bLF	0.5% of TSP	[159]
*N. benthamiana*	Potexvirus potato virus X (PVX) vector/CaMV 35S	hLF (N-lobe)	0.6% of TSP	[160]
*N. tabacum* cell culture	pCAMBIA 1301/CaMV 35S	hLF	0.7–2.7% of TSP	[161]
*Nicotiana tabacum xanthi*	pBIOC21/CaMV 35S	hLF	0.1 to 0.3% of TSP	[162]
*N. tabacum*	pAM1400/CaMV 35S	hLF	0.1 to 0.8% of TSP	[163]
*N. tabacum*	pBI121/CaMV 35S	hLF	0.1–0.3% of TSP	[164]
*N. tabacum*	pBI121/CaMV 35S	hLF	1.8% of total cellular protein	[165]
3. *Solanum tuberosum*				
*Solanum tuberosum*	pBIN35LF vector/CaMV 35S	hLF	0.05% of TSP	[166]
*S. tuberosum*	Auxin-inducible manopine synthase (mas) P2 promoter/CaMV 35S	hLF	0.01–0.1% of TSP	[167]
4*. Lycopersicon esculentum*				
*Lycopersicon esculentum*	pBI121/CaMV 35S	hLF	0.5% of TSP	[168]
*L. esculentum*	pBI121/CaMV 35S	hLF	0.1% of TSP	[169]
5*. Pear* (*Pyrus* sp.)				
*Pear* (*Pyrus* sp.)	pBI121/CaMV 35S	bLF	0.3% of TSP	[170]
6*. Panax ginseng*				
Siberian ginseng plant (*Acanthopanax senticosis*)	PCAMBIA2300/oxidative stress-inducible peroxidase (SWPA2) promoter	hLF	3.6% of TSP	[171]
Korean ginseng cell line (*Panax ginseng*)	pCAMBIA/oxidative stress-inducible peroxidase (SWPA2) promoter	hLF	3% of TSP	[172]
7*. Triticum aestivum* (Wheat)				
*Triticum aestivum*	pAM4424/CaMV 35S	bLF	21 to 67 ng/mg tissue	[173]
8. Sweet potato				
*Ipomoea batatas* (cell culture)	Binary vector pLSM1/ CaMV 35S	hLF	3.2 µg/mg (total protein)	[174]
9*. Hordeum vulgare* (Barley)				
Three commercial cultivars of barley (Oksamytoviy, Vodogray, Hetman)	pHLFTuBA vector/ Rice glutelin B-1 (GluB-1) promoter	bLF	0.5–1.2% of TSP	[175]
*Hordeum vulgare*	pAHC25/Ubiquitin (Ubi) promoter.	hLF	3 ng/mg of TSP	[176]
10*. Alfalfa*				
Alfalfa (*Medicago sativa*)	pBI/CaMV 35S	hLF	0.0047% of TSP	[177]
11*. Edible algae*				
*Chlamydomonas reinhardtii*	pCAMBIA1301C/ CaMV 35S	hLF	1.82% of TSP	[178]
*Chlorella vulgaris* (green algae)	pCAMBIA1304/ CaMV 35S	bLF (N-lobe)	0.5% of TSP	[179]

LFcinH: human lactoferricin; TSP: total soluble protein.

**Table 10:** Comparative analysis of lactoferrin expression systems.

Expression System	Range of Expression (mg/mL)	Advantages	Disadvantages
1. Prokaryotic expression			
Bacteria	0.002–10.6	Highly optimized, short production timeline	Lack of glycosylation, Inclusion body formation
2. Eukaryotic expression			
Yeast	0.00027–3.5	High density fermentation, glycosylated proteins	High rate of translocation, N-glycosylation
Filamentous fungi	0.005–2	High yield, proper protein folding, and mammal like glycosylation patterns.	Improper glycosylation, proteolytic degradation of expressed protein.
Insect and insect cell line	0.0095–10	High expression levels, better post-translation modifications than bacteria	Non-human glycosylation, high production costs
Mammalian cell culture	0.0006–1.6701	Accurate glycosylation and proper protein folding	High production cost and lower yields compared to other systems
3. Transgenic animals			
Cattle	0.0098–13.6 (milk)	Cost effective and enhanced nutritional value	Animal welfare and ethical issues
Goat	0.765–30 (milk)	Large scale, cost-effective production system	Animal welfare and ethical issues
Swine	6.5 (milk)	Dual animal model such as bioreactor and human disease models	Low milk yield, animal welfare issues
Rabbits	0.2–2.3 (milk)	High reproductive rate and fast maturation, efficient bioreactor	Lower milk yield compared to larger animals
Mice	0.0000001–30 (milk)	High reproducibility, cost-effective genetic studies	Low expression levels in milk, high variability in protein expression
Chicken	0.1–0.3 (culture)	Fast growth, high reproductive rate, easy to handle	Ethical concerns with genetic modifications
4. Transgenic plants/crops			
Rice	0.05–100%	High scalability, easy to harvest in large quantities	Environmental impact, regulatory issues
Tobacco	0.1–2.7% TSP	Easy genetic manipulation	Potential for allergens
Potato	0.01–0.1% TSP	Cost-effective, easy to grow	Low protein yield compared to other crops
Tomato	0.1–0.5% TSP	Easy genetic manipulation	Low protein yield compared to other crops
Pear	0.3% TSP	High yield, easy to harvest	Environmental concerns
*Panax ginseng*	3–3.6% TSP	High potential for pharmaceutical applications	Low yield in controlled environments
Wheat	21 to 67 ng/mg tissue	Highly abundant, high transformation efficiency	Lower protein concentration
Sweet potato	3.2 µg/mg (total protein)	High yield, easy to grow	Lower protein concentration compared to other crops
Barley	0.0003–1.2% TSP	High yield, adaptable to a variety of environmental conditions	Low protein concentration, difficult to scale up.
